# Variations in lower limb alignments indicate pelvic tilt after total hip arthroplasty

**DOI:** 10.1186/s12891-022-06032-y

**Published:** 2022-12-22

**Authors:** Kangming Chen, Jinyan Wu, Gangyong Huang, Changquan Liu, Chao Shen, Junfeng Zhu, Yang Li, Tao Li, Fei Xiao, Jianping Peng, Xiuguo Han, Xinhai Zhang, Jun Xia, Xiaodong Chen

**Affiliations:** 1 Department of Orthopaedics, Huashan Hospital, Fudan University, No.12 Middle Wulumuqi Road, Jing’an District, 200040 Shanghai, People’s Republic of China; 2 Department of Orthopaedics, Xinhua Hospital affiliated to Shanghai Jiao Tong University School of medicine, No.1665 Kongjiang Road, Yangpu District, 200092 Shanghai, People’s Republic of China; 3 Present address: Graduate School of Peking Union Medical College and Chinese Academy of Medical Sciences, No. 9 Dongdan Santiao, Dongcheng District, 100730 Beijing, China; 4 Present address: Department of Orthopaedic Surgery, China-Japan Friendship Hospital, No. 2 Yinghuayuan East Street, Chaoyang District, 100029 Beijing, China

**Keywords:** Spinal sagittal balance, Spinopelvic mobility, Total hip arthroplasty, Instability, Lower limb alignment

## Abstract

**Objective:**

We sought to correlate various spinopelvic and lower limb alignments, and to examine the current spinopelvic theories on a Chinese cohort.

**Methods:**

We retrospectively reviewed 166 patients undergoing THA. Among them, 138 patients with unilateral THA met the inclusion criteria. Sagittal alignments and cup orientations were measured on standing and sitting lateral EOS images. Patients were categorized into two groups with a scoring system for lumbar spine degeneration. Patients’ demographics including age, sex, lumbar spine degeneration and radiographic measurements were studied.

**Results:**

PT, SS, LL and TK differed significantly between standing and sitting within each group except for TK in degenerative group (32.8 ± 13.9 vs. 32.9 ± 14.2, *p* = 0.905). Compared with degenerative spine group, non-degenerative spine patients have great pelvic mobility (ΔPT, -24.4 ± 12.5° vs. -17.6 ± 10.7, *p* = 0.0008), greater lumbar mobility (ΔLL, -34.8 ± 15.2 vs. -21.7 ± 12.2, *p* = < 0.0001) and compensatory cup orientation changes (ΔRA, -15.5 ± 11.1 vs. -12.0 ± 8.4, *p* = 0.00920; ΔRI, -10.8 ± 11.5 vs. -5.6 ± 7.5, *p* = 0.0055). Standing PT and ankle dorsiflexion angle correlated positively (R^2^ = 0.236, *p* = 0.005).

**Conclusion:**

THA patients in this cohort showed a spinopelvic motion paradigm similar to that from previous studies on Caucasians. Ankle dorsiflexion indicate greater posterior pelvic tilt on standing. Surgeons should beware of risks of instability in patients with lower limb compensations.

**Advances in knowledge:**

This study provides new insights into the clinical relevance of lower limb alignments to spinopelvic motion after THA in a relatively young Chinese population.

**Supplementary Information:**

The online version contains supplementary material available at 10.1186/s12891-022-06032-y.

## Introduction

The idea of safe zone for acetabular component orientation was proposed over 40 years ago by Lewinnek et al [1], which has guided hip surgeons for long. However, the past ten years witnessed the increasing challenges to the Lewinnek safe zone [2, 3]. A majority of authors attributed the unexpected hip instability to abnormal spinopelvic motions.

Using EOS imaging system, Lazennec [4] should be credited to his pioneering work on the spine-hip relationship. This low-dose biplanar imaging system enabled the authors to explore the spine-hip relationship in various body positions without exposing participants to greater radiation [5]. For normal spine-pelvis-hip motion, pelvis tilts forwards when patients stands and tilts backwards when patients sit [6]. Since the acetabulum/acetabular component is part of the pelvis, the former tilts simultaneously in a proportional extent to the pelvis tilt. Hence, forward tilt of the pelvis serves as a protective mechanism against posterior impingement and anterior instability with patients standing while backward tilt prevents anterior impingement and posterior instability when patients sit.

Although much effort has been taken on spine-hip relationships in elder Caucasians [7–9], to date, hardly could we find any study on a Chinese population including patients with a younger age. The growing number of THAs performed each year in China highlights that the importance of appropriate acetabular component positioning [10]. What’s more, the majority of studies merely focused on spinopelvic alignments although EOS offers more the upper part of human bodies. This study was aimed to verify the established spine-hip relationship theories in a Chinese population with a wide range of age, and to explore the correlation between various sagittal alignments to the pelvic orientation.

## Materials and methods

**Fig. 1:**
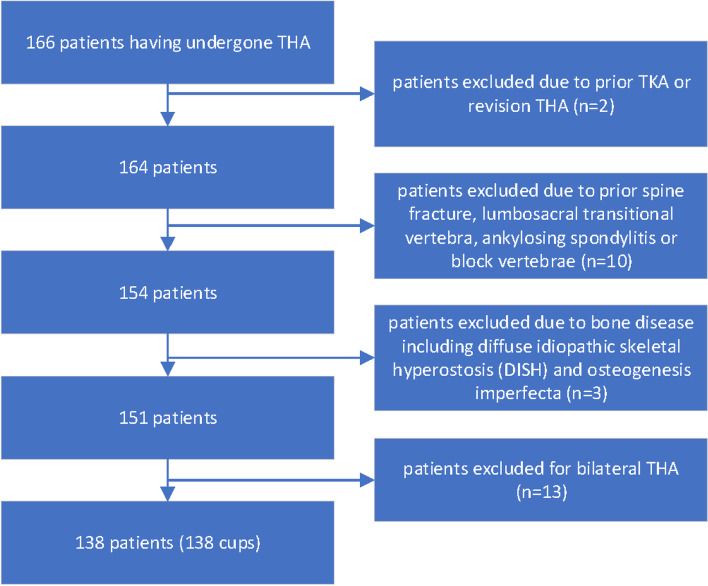
The flow chart depicting the patient inclusion and exclusion process. One hundred and sixty-six THA patients were considered at the initial stage. Patients not suitable for this study were ruled out in a stepwise fashion. At the last stage, 138 patients with unilateral THA performed were finally included

**Fig. 2:**
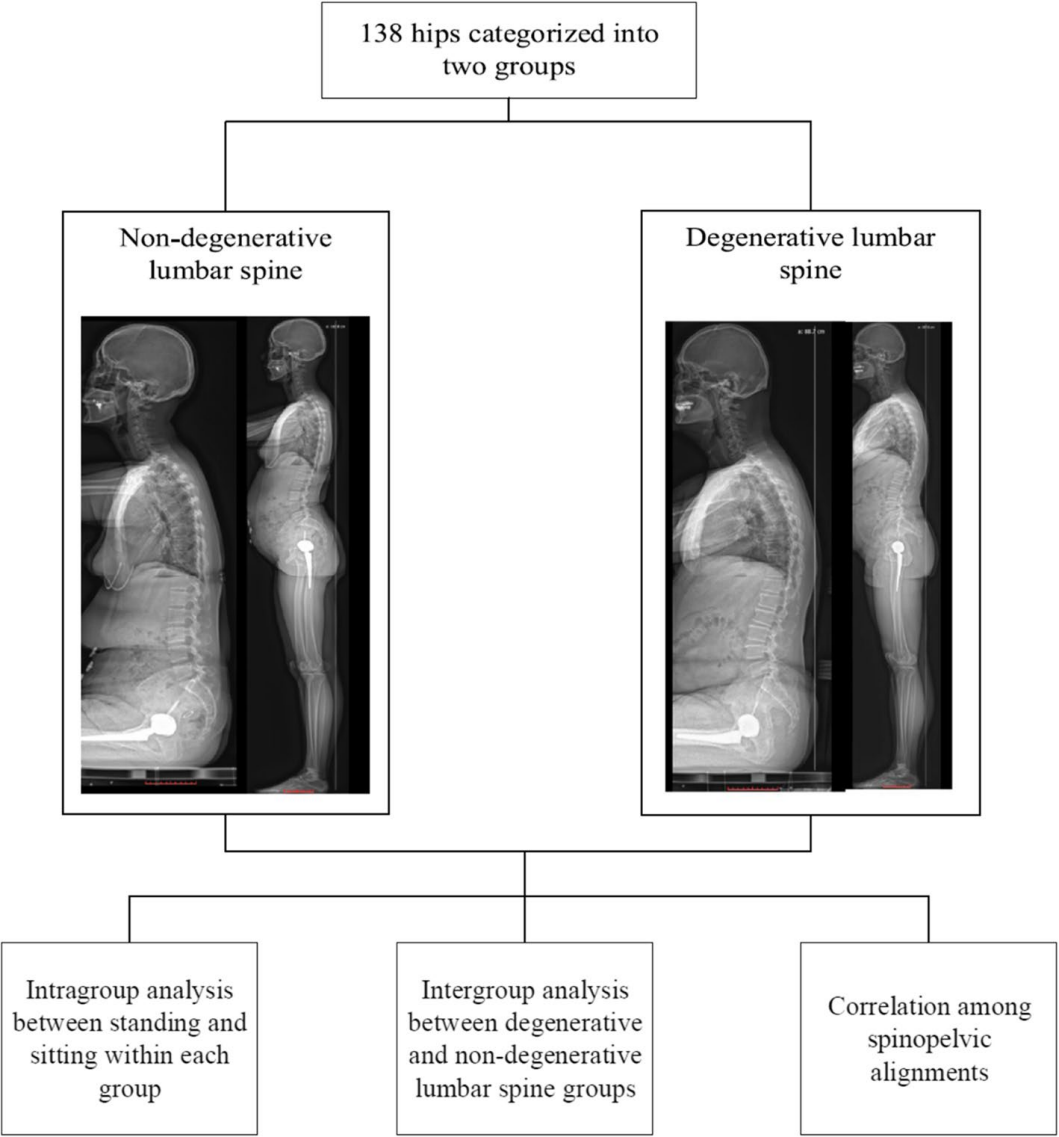
The flow diagram illustrate that after the initial inclusion and exclusion stages, patients were further categorized into non-degenerative and degenerative lumbar spine groups. Intragroup analyses between two postures, and intergroup analyses were conducted, followed by correlation analyses

From June 2018 to August 2019, a consecutive series of 166 outpatients were retrospectively reviewed in whom 138 were included in this IRB-approved study. Informed consent was obtained from all individual participants included in the study. All patients had undergone primary THA performed by one senior surgeon at our institute. They were followed from clinic at 6, 12, 24 weeks and then annually after surgery. The inclusion criteria were as follows: (1) Patients aged over 18 years; (2) having undergone primary unilateral THA; (3) with a diagnosis of femoral neck fracture, developmental dysplasia of the hip, avascular necrosis of the femoral head or Perthes’ disease on one hip. Exclusion criteria were: (1) revision THA; (2) incomplete image acquisition or collection; (3) bilateral THA; (4) bone diseases including diffuse idiopathic skeletal hyperostosis (DISH) and osteogenesis imperfecta; (5) prior spine fracture, lumbosacral transitional vertebra, ankylosing spondylitis or block vertebrae (Figs. 1 and 2).

Posterolateral approach was adopted for these THA patients. Patients were positioned in a lateral decubitus position. All cups were positioned freehand with jigs. We followed the Lewinnek safe zone in which 15°±10° of anteversion and 40°±10° of inclination were suggested for cup orientation [1]. All acetabular and femoral components were cementless. After implanting the components, the surgeon performed impingement test to examine stability.

### Image acquisition

All patients had lateral EOS full-body images (EOS Imaging System; EOS Imaging Inc, Paris, France) in standing and relaxed-seated position at the second-year follow-up. When standing, they lay their hands gently on a bar in front of them and stood still relaxed. Such a posture enabled patients to keep balanced while standing, and minimized the influence of upper limbs on spinal sagittal balanced. Then, patients were asked to sit on a stool and slightly extend their knees to achieve an approximate flat thigh at ease.

### Radiographic measurements

We measured spinopelvic tilt (SPT or simply PT), sacral slope (SS), pelvic incidence (PI), sagittal vertical axis (SVA), lumbar lordosis (LL), thoracic kyphosis (TK) and proximal femoral angle (PFA) for all patients in both standing and sitting positions. Knee flexion angle (KF) and ankle dorsiflexion (AF) were measured for standing images. Operative anteversion (OA) and operative inclination (OI) were measured on lateral standing and sitting EOS images and translated into Murray’s radiographic definitions [11]. We deduced the formulae for correcting horizontal scaling of cup projections on lateral EOS (See supplementary materials). The calculations were performed with MATLAB (MathWorks Inc., Natick, Massachusetts, US).

### Defining spinopelvic alignments

PT is the angle subtended by the vertical line and the line joining the center of bixcoxofemoral axis and the midpoint of S1 endplate. SS is the angle between S1 endplate and the horizontal line. PI is the angle subtended by the perpendicular to S1 endplate and the line connecting the midpoints of S1 endplate and bicoxofemoral axis. LL is the angle between the L1 upper endplate and S1 endplate. TK is the angle between T1 upper endplate and L1 upper endplate. PFA is the angle between the most conspicuous anterior cortex of proximal femur and the vertical. KF (knee flexion angle) is defined as the angle between the mechanical axes of femur and tibia; AF (ankle dorsiflexion) is the angle between the mechanical axis of tibia and the vertical.

Lumbar spine mobility was defined as the change of lumbar lordosis between sitting and standing positions ($\varDelta LL$). Thoracic spine mobility was defined as the change of thoracic kyphosis between standing and sitting positions ($\varDelta TK$).

$$\varDelta LL={LL}_{sitting}-{LL}_{standing}$$

$$\varDelta TK={TK}_{standing}-{TK}_{sitting}$$

Pelvic mobility was defined as the degree of pelvis’ rotation around bicoxofemoral axis in relation to the lateral EOS detector in this study. We chose changes in PT ($\varDelta PT$) between standing and sitting to depict pelvic mobility. Hip mobility is equivalent to femoroacetabular flexion which was described with the change of proximal femoral angle ($\varDelta PFA$) between standing and sitting in this study.

$$\varDelta PT={PT}_{standing}-{PT}_{sitting}$$

$$\varDelta PFA={PFA}_{standing}-{PFA}_{sitting}$$

### Defining lumbar disc degeneration

Patients were categorized into two groups: non-degenerative lumbar spine and degenerative lumbar spine. A lumbar spine was defined as ‘non-degenerative’ once at least 1 disc with height loss of over 50%, facet arthrosis or spondylolisthesis was observed on lateral EOS images acquired at clinic visit. Facet arthrosis and spondylolisthesis were identified according to Buckland’s definitions [12] to detect lumbar spine degeneration.

### Statistical analysis

We used R (version 4.1.1) for all the statistical analyses. Shapiro-Wilk test and Royston tests were applied for univariate and bivariate normality, respectively. For homoscedasticity, we chose Levene test. Student’s t tests and Mann Whitney U tests were performed for interclass alignments. Paired t test and Wilcoxon signed rank test were performed to detect the difference among interclass variables. Correlation coefficients among potentially relevant sagittal alignments were calculated with either Pearson or Spearman correlation.

## Results

### Patient demographics

One hundred and thirty-eight patients undergoing unilateral THA were included for sagittal spinopelvic and lower limb alignments analysis. Bilateral THA patients were excluded, for they constituted only about one-tenth of the population. Ruling them out would not influence statistical significance but rather enable us to use statistical models with greater power (Table 1).

**Table 1:** Demographics

	Non-degenerative spine	Degenerative spine
Age(years)^a^	39.8 ± 14.2	66.1 ± 9.1
Sex (female/male)	62/19	38/19

^a^Data are expressed as mean ± standard deviation

### Intraclass analysis of spinopelvic alignments

PT, SS, LL and TK differed significantly between standing and sitting within each group except for TK in degenerative group (32.6 ± 13.6 vs. 32.7 ± 13.2, *p* = 0.791) (Table 2).

**Table 2:** Intraclass comparison of sagittal alignments from sitting to standing

Sagittal Alignments	Non-degenerative Spine			Degenerative Spine		
	Sitting	Standing	*p* value	Sitting	Standing	*p* value
PT	30.9±10.9	6.5±7.4	<0.001^*^	29.2±12	11.6±9.1	<0.001^*^
SS	17±12.8	40.3±8.7	<0.001^*^	21.2±10.9	37.4±9.9	<0.001^*^
PI	47.9±11.5	46.9±10.9	0.004^*^	50.4±11.7	49.1±11.8	0.029^**^
LL	18.3±16.8	53.1±12.5	<0.001^*^	27.1±16.5	48.8±14.2	<0.001^*^
TK	23.5±11.6	28±10.9	<0.001^*^	32.9±14.2	32.8±13.9	0.905^*^
SVA	58.9±26	2.1±27.4	<0.001^*^	56.8±26.6	20.4±33.9	<0.001^*^
PFA	81.9±13.3	4.3±5.4	<0.001^**^	85.5±4.6	7.2±5.6	<0.001^*^
KF	-	0.8±6.2		-	-2.8±8.6	
AF	-	5.1±3.3		-	6.8±4.4	

Significance level, $\alpha$, was set at 0.05. Data were expressed as mean ± standard deviation

*PT* Spinopelvic tilt or pelvic tilt, *SS* Sacral slope, *PI* Pelvic incidence, *LL* Lumbar lordosis, *TK* Thoracic kyphosis, *SVA* Sagittal vertical axis, *PFA* Proximal femoral angle, *KF* Knee flexion angle, *AF* Ankle dorsiflexion angle

*p values from paired t test

**p values from Wilcoxon signed rank test

### Interclass analysis of sagittal alignments

Non-degenerative spine patients had greater pelvic mobility as shown by larger ΔPT with statistical significance (-24.2 ± 12.5° vs. -17.5 ± 10.4, *p* = 0.0005). So were lumbar mobilities of which less radiographically degenerative spine had greater lumbar mobility (ΔLL, -34.7 ± 15.2 vs. -21.5 ± 12.2, *p* = < 0.0001). Compared with degenerative spine patients, non-degenerative spine patients showed greater change in RA (-15.2 ± 10.8 vs. -11.9 ± 8, *p* = 0.0278) and RI (-10.5 ± 11.3 vs. -5.5 ± 7.3, *p* = 0.0009) from sitting to standing (Table 3).

**Table 3:** Interclass comparison of sagittal alignments and cup orientations

	Non-degenerative Lumbar Spine	Degenerative Lumbar Spine	*p* value
ΔPT	-24.4 ± 12.5	-17.6 ± 10.7	0.0008^*^
ΔSS	23.3 ± 12.9	16.3 ± 11.7	0.0012^*^
ΔLL	-34.8 ± 15.2	-21.7 ± 12.2	< 0.0001^*^
ΔTK	4.5 ± 6.7	-0.1 ± 4.7	< 0.0001^*^
ΔSVA	-56.8 ± 31.6	-36.4 ± 28.6	0.0001^*^
ΔPFA	-77.6 ± 13.1	-78.3 ± 6.9	0.4877^**^
ΔRA	-15.5 ± 11.1	-12 ± 8.4	0.0920^**^
ΔRI	-10.8 ± 11.5	-5.6 ± 7.5	0.0055^**^
AF	0.8 ± 6.2	-2.8 ± 8.6	0.0141^**^
KF	5.1 ± 3.3	6.8 ± 4.4	0.0268^**^

Significance level,$\alpha$, was set at 0.05. Data were expressed as mean ± standard deviation

‘Δ’ refers to changes from sitting to standing. *PT* Spinopelvic tilt or pelvic tilt, *SS* Sacral slope, *PI* Pelvic incidence, *LL* Lumbar lordosis, *TK* Thoracic kyphosis, *SVA* Sagittal vertical axis, *PFA* Proximal femoral angle, *RA* Radiographic anteversion, *RI* Radiographic inclination, *KF* Knee flexion angle, *AF* Ankle dorsiflexion angle

*p values from Student’s t test

**p values from Mann-Whitney U test

### Correlation among sagittal alignments

Lumbar mobility between the two postures was correlated with pelvic mobility positively (*R*^*2*^ = 0.874, *p* < 0.001). Of note, standing SVA was correlated with both knee flexion (*R*^*2*^=-0.369, *p* < 0.001) and ankle dorsiflexion angle (*R*^*2*^ = 0.232, *p* = 0.006). Standing PT and ankle dorsiflexion angle correlated positively (*R*^*2*^ = 0.236, *p* = 0.005) (Tables 4 and 5).

**Table 4:** Correlation coefficient

	ΔTK	ΔLL	ΔSS	ΔPT	KF	AF	PI	Standing PT	Standing SVA
ΔTK	-	-0.200	-0.067	0.087	0.428	-0.323	-0.024	-0.054	-0.337
ΔLL	-	-	-0.916	0.874	-0.116	0.193	0.034	0.485	0.284
ΔSS	-	-	-	-0.954	-0.084	-0.0423	0.024	-0.426	-0.063
ΔPT	-	-	-	-	0.009	0.084	0.026	0.445	0.066
KF	-	-	-	-	-	-0.887	-0.093	-0.108	-0.369
AF	-	-	-	-	-	-	0.074	0.236	0.232
PI	-	-	-	-	-	-	-	0.589	0.307
Standing PT	-	-	-	-	-	-	-	-	0.379
Standing SVA	-	-	-	-	-	-	-	-	-

**Table 5:** *P* value

	ΔTK	ΔLL	ΔSS	ΔPT	KF	AF	PI	Standing PT	Standing SVA
ΔTK	-	0.019^*^	0.435^*^	0.308^*^	< 0.001^**^	< 0.001^*^	0.782^**^	0.532^*^	< 0.001^**^
ΔLL	-	-	< 0.001^*^	< 0.001^*^	0.174^**^	0.024^*^	0.692^*^	< 0.001^*^	0.001^**^
ΔSS	-	-	-	< 0.001^*^	0.328^**^	0.622^*^	0.776^*^	< 0.001^*^	0.463^**^
ΔPT	-	-	-	-	0.913^**^	0.329^*^	0.759^**^	< 0.001^*^	0.439^**^
KF	-	-	-	-	-	< 0.001^**^	0.280^**^	0.208^**^	< 0.001^**^
AF	-	-	-	-	-	-	0.388^**^	0.005^*^	0.006^**^
PI	-	-	-	-	-	-	-	< 0.001^*^	< 0.001^**^
Standing PT	-	-	-	-	-	-	-	-	< 0.001^**^
Standing SVA	-	-	-	-	-	-	-	-	-

‘Δ’ refers to changes from sitting to standing. *PT* Spinopelvic tilt or pelvic tilt, *SS* Sacral slope, *PI* Pelvic incidence, *LL* Lumbar lordosis, *TK* Thoracic kyphosis, *SVA* Sagittal vertical axis, *KF* Knee flexion angle, *AF* Ankle dorsiflexion angle

*p values from Pearson correlation

**p values from Spearman correlation

## Discussion

Visions have been raised up among hip surgeons for the influence of spinopelvic alignments on acetabular component in THA in the past 5 years or so [4, 13–15]. The hypotheses were validated by numerous studies that limited spinopelvic motion would lead to component impingement and instability. However, until now, there is still a lack of studies on whether and to what extent spinopelvic motions affect Chinese patients undergoing THA in both the young and older populations. To our knowledge, this study is among the very first to bridge the gap of ethnicity and age, and to explore the clinical relevance of lower limb alignments in the field of THA and spinopelvic motion.

Normally, a pelvis tilts backward along the bicoxofemoral axis from standing to sitting, which allows clearance for the proximal femur and protect against posterior instability. Conversely, pelvis’s tilting forward from sitting to standing leaves space for the posterior proximal femur and prevent anterior instability. This is what the authors called normal spinopelvic motion [6]. Normal spinopelvic motion depends on a flexible and balanced spine. A rigid lumbar spine lacks enough mobility to accommodate for posterior pelvic tilt during sitting [16]. An unbalanced spine would call upon compensatory mechanisms to restore spine sagittal balance including posterior pelvic tilt. Such compensation is a double-edged sword because posterior pelvic tilt bridges the gap between the posterior proximal femur and acetabular component. This facilitates anterior instability of the components.

Our data support the established theories of spinopelvic motion on elder Caucasians [17]. Both non-degenerative spine and degenerative spine patients showed significant change of LL, SS, PT from sitting to standing. We did not detect a difference of TK in degenerative spine, which is in line with the rigid nature of degenerative spines. PFA between postures differ significantly while ΔPFA do not between the two groups. This means overall changes between two body positions were similar, whether the patient has a non-degenerative or degenerative spine. Such comparable body position changes between groups laid a solid foundation for further analysis.

Patients with a non-degenerative spine has greater lumbar mobility and pelvic mobility, as shown by larger ΔLL and ΔPT. Also, non-degenerative spine patients have greater ΔRA and ΔRI. Since acetabular components was a part of the pelvis, changes in pelvic tilt would be accompanied by operative anteversion of the same degrees [17] due to the equivalence of ante-inclination and operative anteversion. Based on Murray’s piece of cup orientation definitions, we can easily deduce that changes in one definition of cup alignments means change in the other definitions, but usually not of the same degrees [11]. Hence, in the next step, we looked for the correlations among PT, ΔPT and other alignments since PT connects spinopelvic motion and cup alignments.

Positive correlation between ΔPT and ΔLL further verified that the greater lumbar mobility, the greater pelvic mobility. Furthermore, lower limb alignments offered us a new perspective on how to identify a patient at risk of cup instability. A larger SVA means a spine moves toward unbalanced state during which posterior pelvic tilt, knee flexion and ankle dorsiflexion may be called upon. The positive correlation between standing PT and ankle dorsiflexion (*R*^*2*^ = 0.236, *p* = 0.005) suggest that the latter be an indicator of posterior pelvic tilt during standing. Under such circumstances, surgeons should be wary of the risk of too much cup anteversion leading to posterior impingement and anterior instability after THA. These patients correspond to the ‘unbalanced types’ in Phan’s categorization of spinopelvic motion in postoperative THA patients [16].

This study has its strength. First, inclusion of both young and elder patients enables us to extrapolate the present theory established from much older populations to a wider range of age. In this cohort, DDH with frank dislocation or in arthritic stage, and avascular necrosis of femoral head (AVN) in the late stage are the two major indications for THA in young adults. In 2017, a cross-sectional study of 29,180 participants estimated the overall prevalence of DDH to be 1.52% in China [18]. After stratification with age, young participants showed an even higher-than-overall prevalence. This highlights the external validity required for studies to have enough power to work well on a wider range of age, especially the young. Second, limb alignments can be an easy and useful sign to find out potential at-risk patients at clinic. For young adults whose knee and ankle joints usually have full passive range of motion, clinicians should suspect slight knee flexion or ankle dorsiflexion as compensatory mechanisms for spinal sagittal balance. Although compensations taken may vary among various individuals, the general rule is that posterior pelvic tilt is most common strategy [19]. Any involvement to less frequent knee and ankle joints indicates increased SVA, a spinal sagittal balance measure, which may in turn indicate greater posterior pelvic tilt theoretically. Our data confirmed the hypothesis on the ankle aspect with a positive correlation between standing PT and ankle dorsiflexion.

There are limitations on this study. The population is small at a single institute compared with previous studies conducted in countries with registration systems [2]. What’s more, this is a retrospective study on post-operative patients. Hip mobility may change after surgery, which would in turn affect the sagittal spinal balance. Further studies including pre-operative data are warranted in an attempt to predict the post-operative cup alignments even before surgery. Furthermore, we measurement PI on both positions and noticed that standing and sitting PI were significantly different. This may be attributed to the slight difference in pelvic orientation when patients change their body postures during image acquisition. However, approximately 1° of overall difference does not compromise the validity despite statistical significance.

## Conclusion

To our knowledge, this is the first study on the influence of spinopelvic motion on both young and old Chinese patients undergoing THA. The spinopelvic motion theory works on Chinese patients as well. Of note, we proposed the lower limb alignments be indicators for abnormal spinopelvic motion which may bring about suboptimal outcomes after THA. Further investigations regarding both pre- and post-operative alignments are warranted.

## Supplementary Information

**Additional file 1.** Correcting horizontal distortions of cups on lateral EOS films.

## Data Availability

The datasets used and/or analyzed during the current study are available from the corresponding author on reasonable request.
