# Supplementary material for: Variations in lower limb alignments indicate pelvic tilt after total hip arthroplasty
Source: BMC Musculoskelet Disord. 2022 Dec 22;23:1116. doi: 10.1186/s12891-022-06032-y (PMC9773480; doi:10.1186/s12891-022-06032-y)
Supplement: Supplementary file 1 — Additional file 1. Correcting horizontal distortions of cups on lateral EOS films. [file 12891_2022_6032_MOESM1_ESM.docx]

**Correcting horizontal distortions of cups on lateral EOS films**

Suppose the semi-minor axis of a horizontally scaled ellipse,$C_{OE2}$, is $m$, semimajor axis being $n$ and the inclination of semimajor axis being $\beta$. Its corresponding ellipse before horizontal scaling, $C_{OE}$, has a semi-minor axis, $a$, a semimajor axis, $b$, and inclination of semi-major axis, $\alpha$. (Supplementary figure 1)

$m$, $n$ and $\beta$ determine $C_{OE2}$ and can be measured on lateral EOS images by using a validated software, Surgimap (Nemaris Inc., New York, NY, US). To obtain $C_{OE}$ is to scale the $C_{OE2}$ by a reverse scaling factor,$k^{-1}$. $k$ can be derived from the horizontal axis, $m_{0}$ and the vertical axis, $n_{0}$ of the ellipse where the whole cup image lies. $m_{0}$ and $n_{0}$ can be measured on lateral EOS images. We get

$$\begin{aligned} k=\frac{m_{0}}{n_{0}}\#(1-1) \end{aligned}$$

And the reverse scaling matrix is written as

$$\begin{aligned} \left( \begin{matrix} \frac{1}{k} & 0 \\ 0 & 1 \end{matrix} \right)\#(1-2) \end{aligned}$$

Plug the reverse scaling matrix into $C_{OE2}$ and then we get the equation of $C_{OE}$

$$\begin{aligned} \left( \frac{xkcos\beta+ysin\beta}{m} \right)^{2}+\left( \frac{-xksin\beta+ycos\beta}{n} \right)^{2}=1\#(1-3) \end{aligned}$$

which can be written in the general form. Thus, the quadratic term coefficients $a_{11}^{'}$, $a_{12}^{'}$ and $a_{22}^{'}$ can be derived. Plug the quadratic term coefficients into the following rotation formula of quadratic curves,

$$\begin{aligned} 2\left( a_{22}^{'}-a_{11}^{'} \right)\sin\alpha\cos\alpha+2a_{12}^{'}\left( \cos^{2} \alpha-{sin}^{2}\alpha\right)=0\#(1-4) \end{aligned}$$

Solve the equation, again with MATLAB, and we get $\alpha$. $\alpha$ is the operative anteversion before correction of pelvic obliquity and pelvic rotation, which is denoted by $OAi$.

$$\begin{aligned} OAi=\alpha\#\left( 1-5 \right) \end{aligned}$$

Let $A$ be the matrix of quadratic terms of equation (1-4).

$$\begin{aligned} A=\left| \begin{matrix} a_{11}^{'} & a_{12}^{'} \\ a_{12}^{'} & a_{22}^{'} \end{matrix} \right|\#\left( 1-6 \right) \end{aligned}$$

Calculate the two eigenvalues of $A$, $\lambda_{1}$ and $\lambda_{2}$ in which $\lambda_{1}<\lambda_{2}$. The ratio of the semi-minor axis to the semi-major axis of $C_{OE}$ is thus $\sqrt{\frac{\lambda_{1}}{\lambda_{2}}}$. Adapting Lewinnek’s method[1] to lateral EOS images, we have the operative inclination $OIi$.

$$\begin{aligned} OIi={sin}^{-1} \sqrt{\frac{\lambda_{1}}{\lambda_{2}}}\#\left( 1-7 \right) \end{aligned}$$

**Converting to radiographic definitions**

Radiographic anteversion (RA) and radiographic inclination (RI) can be easily derived with trigonometry equations proposed by Murray[2],

$$\begin{aligned} \sin\left( RA \right)=\frac{\sin\left( OA \right)}{\cos\left( OI \right)}\#\left( 1-8 \right) \end{aligned}$$

$$\begin{aligned} \tan\left( RI \right)=\frac{\tan\left( OI \right)}{\cos\left( OA \right)}\#\left( 1-9 \right) \end{aligned}$$

The above calculations are carried out in MATLAB (MathWorks Inc., Natick, Massachusetts), US.

Reference

1. Lewinnek G, Lewis J, Tarr R, Compere C, Zimmerman J: **Dislocations after total hip-replacement arthroplasties**. *J Bone Joint Surg Am* 1978:217-220.

2. Murray DW: **The definition and measurement of acetabular orientation**. *J Bone Joint Surg Br* 1993, **75**(2):228-232.

Figure


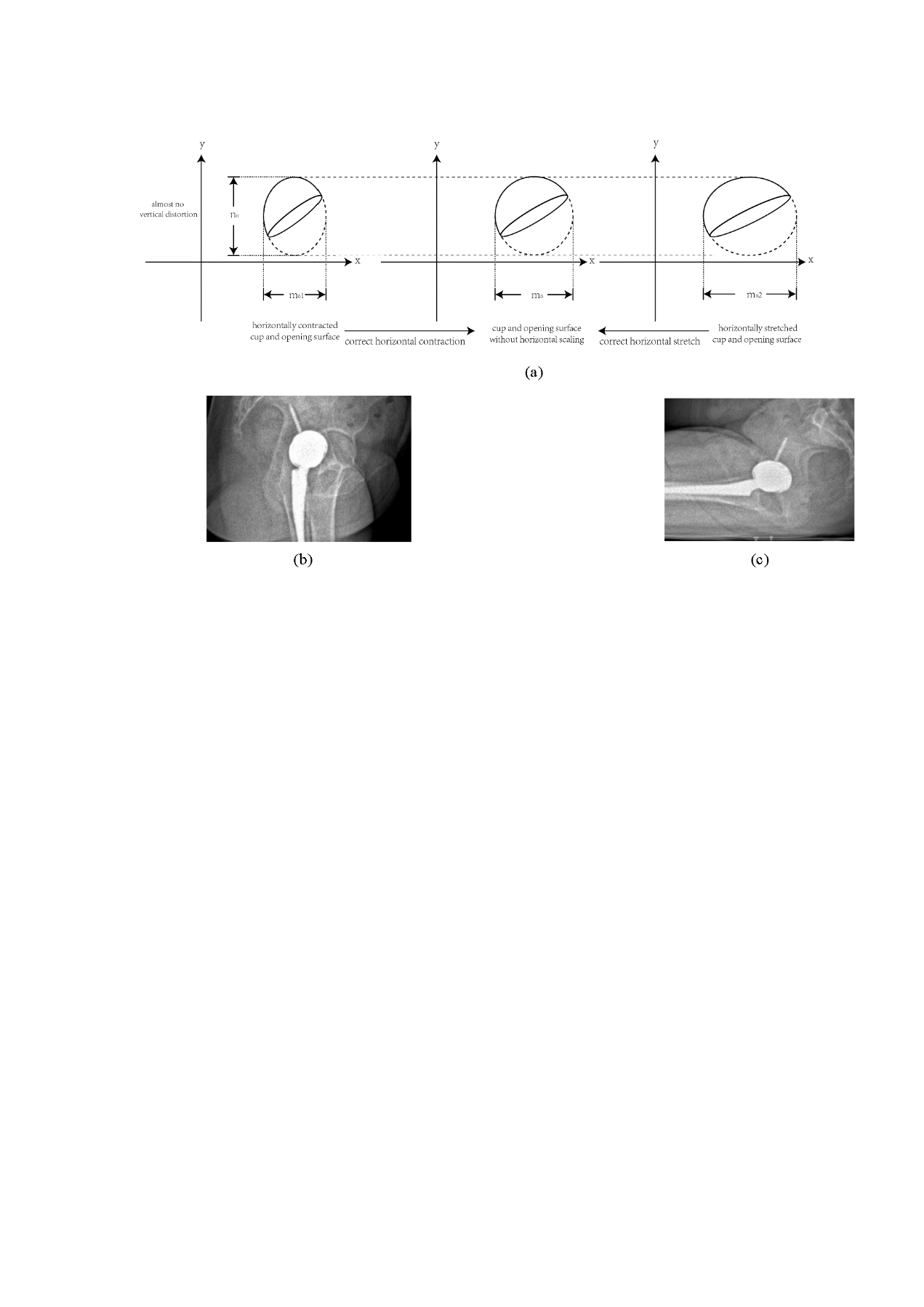


Supplementary figure 1. Schematic diagrams and authentic lateral EOS images showing horizontal scaling of cup images and the correction strategies. (a) Lateral EOS images of the cup and its opening surface are either contracted or stretched horizontally, but almost remain the same in the vertical direction. This results in increased or decreased OA and OI, respectively. The correction strategy is to obtain the scaling factor and measure the horizontally scaled cups, and then construct an unscaled ellipse which depicts the images of the cup and its opening surface before horizontal scaling. (b) A lateral EOS image with a horizontally contracted cup. (c) A lateral EOS image with a horizontally stretched cup.
